# Review of Directed Self-Assembly Material, Processing, and Application in Advanced Lithography and Patterning

**DOI:** 10.3390/mi16060667

**Published:** 2025-05-31

**Authors:** Xiuyan Cheng, Di Liang, Miao Jiang, Yufei Sha, Xiaonan Liu, Jinlai Liu, Qingchen Cao, Jiangliu Shi

**Affiliations:** Beijing Superstring Academy of Memory Technology, Beijing 100176, China; xiuyan.cheng@bjsamt.org.cn (X.C.); di.liang@bjsamt.org.cn (D.L.); yufei.sha@bjsamt.org.cn (Y.S.); xiaonan.liu@bjsamt.org.cn (X.L.); jinlai.liu@bjsamt.org.cn (J.L.); qingchen.cao@bjsamt.org.cn (Q.C.); jiangliu.shi@bjsamt.org.cn (J.S.)

**Keywords:** DSA, advanced lithography technology, BCPs

## Abstract

Directed self-assembly (DSA) lithography, a cutting-edge technology based on the self-assembly of block copolymers (BCPs), has received significant attention in recent years. Combining DSA with established lithography technologies, such as extreme ultraviolet (EUV), deep ultraviolet (DUV), electron beam lithography, and nanoimprint lithography, significantly enhances the resolution of target patterns and device density. Currently, there are two commonly used methods in DSA: graphoepitaxy, employing lithographically defined topographic templates to guide BCP assembly, and chemoepitaxy, utilizing chemically patterned surfaces with precisely controlled interfacial energies to direct nanoscale phase segregation. Through novel DSA lithography technology, nanoscale patterns with smaller feature sizes and higher densities can be obtained, realizing the miniaturization of hole and line patterns and pitch multiplication and improving the roughness and local critical dimension uniformity (LCDU). It is gradually becoming one of the most promising and advanced lithography techniques. DSA lithography technology has been applied in logic, memory, and optoelectronic device fabrications.

## 1. Introduction

The semiconductor industry’s relentless scaling roadmap now confronts critical bottlenecks as conventional photolithography struggles with fundamental resolution limits and prohibitive cost scaling. With feature sizes now penetrating the sub-10 nm regime, the limitations of optical diffraction and escalating costs associated with advanced lithographic techniques threaten the continuation of Moore’s Law. EUV lithography, while enabling 7 nm node production, faces commercialization challenges, such as tool costs exceeding $150 million and complex vacuum system requirements [1]. Concurrently, multi-patterning techniques, while improving resolution, have increased mask counts from 40 layers at 28 nm to over 100 layers at 5 nm nodes, increasing lithography’s cost contribution from 18% to 42% of total wafer fabrication budgets [2]. Physical constraints compound these economic pressures: 193 nm argon fluoride (ArF) immersion lithography approaches its 38 nm Rayleigh resolution limit, while line-edge roughness (LER) remains above 1 nm, despite sophisticated resolution enhancement technologies (RETs) like optical proximity correction [3]. These dual challenges of technical and economic pressures have accelerated the development of next-generation patterning technologies, among which DSA has emerged as a particularly promising candidate [4]. The combination of DSA and DUV realizes line/space and contact hole multiplication. The International Roadmap for Devices and Systems (IRDS) identifies DSA, in combination with EUV, as a next-generation advanced patterning technique [5]. The synergy of DSA with EUV enables sub-10 nm resolution enhancement, defect rectification, and improved roughness while reducing EUV dose requirements by 30–50% through self-aligned pattern multiplication. EUV-defined chemical templates guide DSA to achieve atomic-scale CDU, overcoming the photoresist stochastic limitations of standalone EUV.

At its core, DSA represents a fundamental transformation from conventional top-down patterning to bottom-up nanostructure formation, using the inherent self-assembly properties of BCPs. BCPs are architecturally precise macromolecules consisting of two or more chemically distinct polymer blocks covalently bonded together. When thermally annealed, BCPs undergo microphase separation driven by the Flory–Huggins interaction parameter (χ) that quantifies the incompatibility between polymer blocks. For instance, the widely studied polystyrene-block-poly(methyl methacrylate) (PS-b-PMMA) system separates into periodic nanodomains, with dimensions directly controlled by the degree of polymerization (N) and χ value, enabling a sub-5 nm feature size through high-χ BCPs [6]. Computational modeling plays a pivotal role in optimizing these self-assembly processes, as demonstrated by Gueorguiev’s studies, where density functional theory (DFT) calculations predicted the stability and electronic properties of self-assembled silicon–metal nanowires [7]. Similarly, molecular dynamics simulations and phase-field modeling can guide the design of BCP systems by predicting equilibrium morphologies, defect formation energetics, and interfacial interactions, bridging molecular-scale insights with macroscale pattern fidelity. The self-assembly process follows the energy minimization principle, where interfacial energy between polymer blocks competes with entropy loss from chain stretching, resulting in predictable nanoscale architectures.

**Figure 1:**
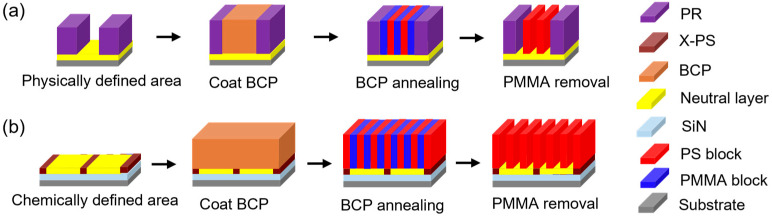
General concept for graphoepitaxy (**a**) and chemoepitaxy (**b**) flows. Adapted from [8].

Graphoepitaxy and chemoepitaxy methods are the two main process strategies for DSA. In physically defined graphoepitaxial processes, phase separation is induced by the selective affinity of one polymer block toward the sidewalls, which act as guiding templates. Meanwhile, the substrates of the confined geometry must exhibit neutral interactions with both blocks (Figure 1) [8]. The critical innovation distinguishing DSA from conventional self-assembly lies in the introduction of chemoepitaxy, a guidance mechanism that imposes long-range order on the otherwise stochastic self-assembly process [9]. This is achieved through pre-patterning substrates with precisely defined chemical or topographic confinements at dimensions larger than the inherent BCP periodicity (L_0_) [10]. These guiding patterns create alternating surface chemistries that direct the orientation of BCP domains. For example, a substrate patterned with alternating hydrophobic and hydrophilic domains can align PS-b-PMMA domains such that the PS block (higher hydrophobicity) preferentially adheres to hydrophobic regions, while PMMA domains occupy hydrophilic areas [11]. This synergistic combination of thermodynamic self-assembly and external guidance enables defect densities 2–3 orders of magnitude lower than purely stochastic self-assembly, with a demonstrated critical dimension uniformity (CDU) of 0.5 nm (3σ) on 300 mm wafers [12]. Advanced guidance schemes further use ternary blends incorporating homopolymers to enhance pattern fidelity and pattern placement error challenges in complex architectures [13].

The self-assembly capability emerges from the dynamic nature of BCP reassembly during annealing. Recent advances in mesoscale modeling, informed by the density functional theory (DFT), have further elucidated how interfacial energetics and diffusion kinetics govern nanostructure formation. For instance, Filho et al. demonstrated a DFT-fed phase-field model (PFM) for semiconductor nanorods, where first-principles-derived interfacial energies and diffusion coefficients predicted core–shell morphologies with compositional gradients analogous to BCP microphase separation [14]. Similarly, Pacheco et al. revealed how cluster integrity in condensed phases depends on the balance between atomic-scale interactions and mesoscale reorganization, a paradigm that directly applies to defect annihilation in BCP systems [15]. Such methodologies exemplify the potential of integrating ab initio insights with mesoscale frameworks to optimize BCP composition diversity (e.g., through χ/N tuning) and defect mitigation (e.g., via annealing kinetics), thereby enhancing DSA’s precision for semiconductor patterning. Unlike photolithography’s static pattern transfer, DSA permits continuous defect annihilation through molecular mobility in the melt state. Kinetic studies reveal that over 90% of dislocation and disclination defects can be eliminated during optimal annealing cycles, a feature impossible to replicate in conventional lithography without multiple exposure and etch steps [16]. This inherent error correction, coupled with the ability to generate 10¹¹ features/cm^2^ in a single processing step, positions DSA as both a complementary enhancement and potential replacement for established lithographic techniques in specific applications, such as contact hole multiplication and fin formation in advanced transistor architectures [17]. DSA utilizes the inherent phase-separation behavior of BCPs to generate periodic nanostructures with adjustable shapes like lamellae, cylinders, and spheres. Under equilibrium conditions, BCPs self-assemble into nanoscale domains (typically 5–50 nm) dictated by their thermodynamic properties, such as Flory–Huggins interaction parameters (χ) and volume fractions. Critically, DSA introduces external guidance, including thermal annealing, surface energy modulation, or external electric/magnetic fields, to direct the orientation of self-assembled patterns. The resulting ordered nanostructures serve as hard masks for pattern transfer, enabling the fabrication of high-density semiconductor devices.

Compared to EUV or nanoimprint lithography (NIL), DSA eliminates the need for expensive light sources, masks, and multi-patterning steps, significantly reducing manufacturing costs while achieving sub-5 nm feature resolution [18]. This fundamental change has attracted substantial interest from academia and industry, with leading institutions, such as EMD, IBM, IMEC, Intel, TEL, and JSR, pioneering material development [19], process optimization, and defect reduction strategies. Concurrently, academic research, including works by groups at UC Berkeley, Fudan University, and Dow Chemical, has advanced the fundamental understanding of BCP thermodynamics, interfacial interactions, and directed alignment mechanisms. As semiconductor nodes approach atomic-scale limits, DSA is expected to complement or even replace the traditional top-down approaches, particularly in applications requiring ultrahigh-density memory, logic devices, and metamaterials [20]. 

## 2. DSA Process Flow Introduction

There are two mainstream process strategies for DSA, namely graphoepitaxy [21] and chemoepitaxy [22]. Both methods enable BCPs to form highly ordered nanostructures over large areas, which has significantly promoted the extensive application of BCPs in lithographic patterning. The fundamental distinction lies in the method of generating the prepattern template for assembly. In the graphoepitaxy method, the BCP assembly region is confined by physical barriers, whereas in the chemoepitaxy method, it is determined by chemical modifications in surface energy on the substrate.

Graphoepitaxy offers advantages such as simple processing, high tolerance, fewer defects, and precise pattern alignment. In contrast, chemoepitaxy is not constrained by the spatial limitations of guiding templates, which contributes to improved pattern quality. However, its limitations lie in the fact that chemical patterning typically requires expensive techniques like EUV lithography or electron beam lithography (EBL), and the process of chemically modifying the substrate surface is highly complex.

### 2.1. Graphoepitaxy Method

The graphoepitaxy method uses lithography techniques (such as optical lithography, EUV lithography, or EBL) to pre-fabricate guiding templates, such as trenches or holes, on the substrate. These templates are utilized to guide the self-assembly of BCPs to form highly ordered nanostructures. The principle is that the template pattern of the substrate imposes structural constraints on the BCPs. The self-assembly of the BCPs is initiated and guided by the sidewalls of the template and then propagated to the central region, achieving a high degree of order for the nanostructures of the BCPs within the entire template. The template pattern width is typically designed as integer multiples of the BCP period.

**Figure 2:**
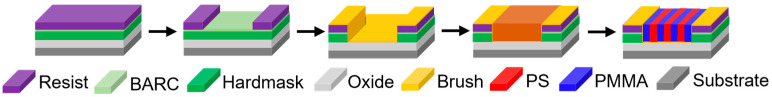
DSA integration scheme with graphoepitaxy. Steps 1 to 3: 193 nm dry lithography line/space patterns creation into a hard mask, which ends with an optional affinity modification process (stripes); steps 4 to 5: BCP coating and self-assembly. Adapted from [23].

The key steps in graphoepitaxy include: (1) guiding pattern fabrication, (2) BCP coating and annealing, (3) selective removal of one block of BCPs, and (4) pattern transfer to the underlying substrate. For example, Oria L. et al. investigated the process optimization of DSA line/space patterning self-assembly using a PS-b-PMMA [23]. The guiding patterns was obtained using 193 nm dry lithography. As shown in Figure 2, when a BCP film is deposited on a substrate with pre-patterned trenches, the BCP is annealed to allow it to separate into two different domains, and the BCP chains tend to align along the trench walls, resulting in a well-ordered pattern. Then, the patterns are transferred by means of dry plasma etching into an organic hard mask constituted of SiARC and SOC. The combination of ArF dry lithography and DSA enables multiple patterning of line/space. Graphoepitaxy is particularly suitable for line/space patterns and has been successfully demonstrated for pitch multiplication applications.

### 2.2. Chemoepitaxy Method

An advantage of the chemoepitaxy method is that it requires less space on the wafer than the graphoepitaxy flows to create the prepatterns.

A chemoepitaxy process involves defining two distinct surface energy regions. The first, known as the guiding region, exhibits a strong preference for one of the blocks in the BCP, serving as a chemical anchor that directs the BCP to phase-segregate into its respective domains during annealing. The second region is designed to be neutral, showing no preferential affinity for either of the blocks in the BCP. This neutrality ensures that the BCP shows perpendicular orientation relative to the substrate surface.

#### 2.2.1. LiNe Flow

**Figure 3:**
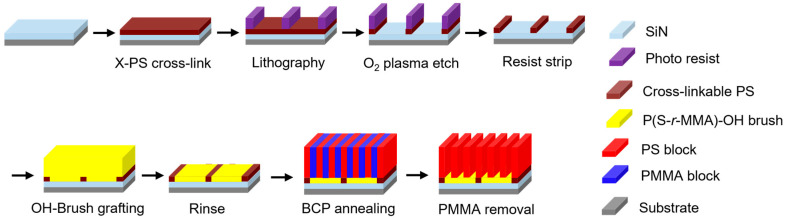
Schematic diagram of the LiNe flow. Adapted from [24].

The LiNe flow begins with the coating of a guiding layer, X-PS (thickness < 10 nm) [24]. As shown in Figure 3, the guiding layer is first patterned using conventional lithography techniques, followed by O_2_ plasma etching. After removing the photoresist, a neutral layer or brush layer (thickness < 10 nm) is coated, then annealed, and the solvent is rinsed to remove excess material on the guiding layer. This brush should adhere to the substrate rather than the guiding layer, creating two regions with different surface affinities for the BCP. Finally, the BCP is coated and thermally annealed to induce the separation of the two BCP blocks. When desired, the PMMA domains are removed with O_2_ plasma. Then, the resulting pattern can be transferred to the underlying substrate.

This process utilizes the chemical contrast between the guiding layer and brush layer to direct the self-assembly of the BCP, enabling the formation of highly ordered nanostructures with precise control over feature size and placement. The LiNe flow process is particularly advantageous for achieving a sub-10 nm feature resolution with reduced defect densities, making it a promising candidate for advanced semiconductor manufacturing.

#### 2.2.2. SMART Flow

**Figure 4:**
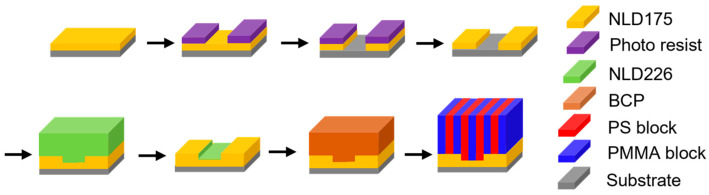
Schematic of SMART flow. Adapted from [25].

As illustrated in Figure 4, the Surface Modification for Advanced Resolution Technology (SMART) process flow is similar to the LiNe flow, but a significant difference is that the neutral layer is patterned first, rather than the guiding layer. The SMART process flow begins with the deposition of the cross-linkable neutral layer NLD-175 on the substrate, subsequently patterned via ArF immersion lithography [25]. Then, the photoresist is stripped away using a resist stripper. Next, a brush pinning material (as a guiding material) NLD-226 is spin-coated onto the patterned neutral layer. Excess pinning material is removed via solvent rinsing, leaving behind a monolayer of pinning material adhered exclusively to the trenches. The process culminates with BCP deposition, followed by thermal annealing, to induce microphase separation and generate well-defined line/space patterns. After selectively etching the PMMA block with O_2_ plasma, the resulting DSA pattern can be transferred to the underlying substrate SiARC film through a plasma etching process. 

The SMART™ flow offers a refined approach to DSA that synergistically combines chemoepitaxy with nanoscale topographic confinement, achieving high-resolution patterning with improved defect control and alignment precision.

#### 2.2.3. Underlayer Surface Treatment Flow

**Figure 5:**
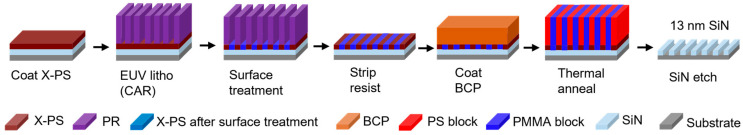
Schematic illustration of the ULST flow for generating L/S patterns. Adapted from [26].

**Figure 6:**
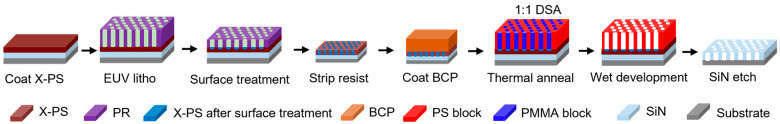
Schematic illustration of the ULST flow for generating hexagonal hole patterns. Adapted from [27].

The underlayer surface treatment (ULST) process flow is described in Figure 5 [26]. This is achieved by treating the underlayer surface to create a pattern of chemical functionality that can guide the DSA of BCPs. Firstly, a 13 nm silicon nitride (SiN) film is coated on a silicon wafer. A 6 nm film of cross-linkable polystyrene (X-PS) is then spin-coated onto the wafer. Then, the guiding pattern is printed using EUV lithography techniques. A surface treatment is used to oxidize the X-PS in the exposed and developed X-PS regions. After removing the remaining photoresist, a BCP of PS-b-PMMA is spin-coated onto the surface and annealed in a low-oxygen atmosphere to drive microphase segregation and facilitate self-assembly. Then, PMMA blocks of BCP are selectively removed, and the final step involves transferring the PS pattern into the 13 nm SiN underlayer by dry etching. The schematic illustration for generating hexagonal holes in Figure 6 is similar to that of creating a line/space pattern in Figure 5 [27].

Both ULST and SMART flows begin with coating a neutral layer, ensuring non-preferential affinity for either of the blocks in the BCP. The key distinction lies in how the guide pattern is formed, with the ULST flow creating the guide pattern through surface treatment UL and the SMART flow achieving this by applying a guiding layer of brush pinning material. Compared to the LiNe and SMART flows, the ULST flow eliminates the need for brush material, thereby removing the steps of coating and rinsing the brush layer and simplifying the overall process flow.

The self-assembly process in graphoepitaxy is directed through the physical confinement of pre-patterned guiding templates, which provides inherent advantages in alignment precision and process simplicity.

Accurate placement of self-assembled patterns at arbitrary wafer positions is enabled by the geometric confinement from guiding patterns, using their spatial constraints to guide domain orientation, while the confinement effect facilitates the fabrication of complex structures. However, the presence of these templates requires sacrificing a portion of the wafer’s surface area for template placement, which limits scalability for high-density periodic array patterns. In contrast to graphoepitaxy, chemoepitaxy employs substrate-selective chemical patterning to guide self-assembly, thereby eliminating spatial limitations while improving long-range order through tailored interfacial energy gradients. This method avoids the need for physical guiding structures, enabling more flexible and uniform patterning. Nevertheless, its limitations include the requirement for expensive techniques like EUV or EBL to achieve precise patterning. Additionally, the process of surface chemical modification is highly complex, involving multi-step treatments to create and maintain the necessary chemical contrast, which complicates scalability and increases production costs.

## 3. Materials for DSA Lithography

### 3.1. Underlayer (UL) and Neutral Layer (NL) Materials

#### 3.1.1. Underlayers (ULs)

ULs serve as the foundational substrate modification layer, engineered to create customized surfaces through precise energy tuning. These layers typically comprise crosslinked polymer matrices or inorganic dielectrics, each class offering unique advantages. Two types of ULs have been extensively explored, including polymeric ULs and inorganic ULs.

##### Polymeric ULs

The industry-standard, crosslinked polystyrene-random-methyl methacrylate (PS-r-MMA, 60-80% styrene) achieves a surface energy (γ) adjustment between 22-28 mN/m through monomer ratio variation [28]. The crosslinking density, controlled via UV or thermal curing (typically 1-5 J/cm^2^ UV dose at 254 nm), must balance mechanical stability with sufficient chemical functionality for subsequent brush layer grafting [29].

##### Inorganic ULs

Silicon-based materials like SiO_2_ (γ ≈ 50 mN/m) and SiN_x_ (γ ≈ 40 mN/m) require surface modification through self-assembled monolayers (SAMs) of alkylsilanes (e.g., octadecyltrichlorosilane) or plasma treatments (CF_4_/O_2_ mixtures) to reduce surface energy into the optimal 20–30 mN/m range [30]. Recent advances employ atomic layer deposition (ALD) of AlO_x_/TiO_x_ nanolaminates (2-5 nm thick) to create gradient surface energies that directionally bias BCP domain orientation [31].

The key to UL performance lies in the suppression of interfacial defects. Excessive hydrophobicity (γ < 20 mN/m) induces lateral phase separation, while insufficient modulation (γ > 30 mN/m) fails to overcome BCP’s inherent interfacial tension. Advanced metrology techniques like X-ray photoelectron spectroscopy (XPS) and contact angle hysteresis analysis (θ_a_/θ_r_ < 5°) are employed to verify UL uniformity at sub-10 nm scales [32].

#### 3.1.2. Neutral Layers (NLs)

NLs constitute the chemically neutral interface between ULs and BCP films, ensuring equal attraction to either polymer block. Modern NLs use precisely designed random copolymer brushes.

These industry-standard brushes of PS-r-PMMA (Mn = 5–10 kg/mol, PDI < 1.2) brushes achieve neutrality through statistical monomer distribution. At a 50:50 PS:PMMA molar ratio, the interfacial energy difference (Δγ = |γ_PS_ − γ_PMMA_|) is minimized to <0.5 mN/m, enabling the perpendicular orientation of lamellar PS-b-PMMA domains with <1° angular deviation [33]. Brush thickness is tightly controlled (3–8 nm) via grafting density (σ = 0.1–0.4 chains/nm^2^), with lower σ values permitting dynamic BCP chain rearrangement during annealing [34].

For high-χ BCPs requiring > 250 °C annealing, thermally stable brushes like hydroxyl-terminated poly(styrene-stat-2-vinylpyridine) (PS-r-2VP-OH) have been developed. The terminal -OH groups (3–5% molar content) form hydrogen bonds with UL surfaces, increasing decomposition temperatures to 280–300°C while maintaining Δγ < 1 mN/m [35]. Emerging “smart” brushes incorporate pH-responsive monomers (e.g., 4-vinylpyridine) that enable post-assembly polarity switching for selective domain removal [29].

NL performance is quantified through neutron reflectometry (NR) and grazing-incidence small-angle X-ray scattering (GISAXS), with state-of-the-art systems achieving >99% perpendicular orientation yield across 300 mm wafers. Recent breakthroughs in photo-patternable NLs enable the direct optical definition of chemical guiding patterns, eliminating the need for separate lithographic steps in DSA flow [36].

### 3.2. Brush and Pinning Materials

Polymer brushes (2–10 nm thick) are covalently grafted via silane (SiO_2_ substrates) or thiol (metal surfaces) coupling chemistry. The grafting density (σ ≈ 0.1–0.5 chains/nm^2^) plays a critical role in BCP self-assembly. Lower σ (<0.2 chains/nm^2^) enhances chain mobility, whereas higher σ (>0.3 chains/nm^2^) induces pronounced pinning effects that effectively stabilize metastable structures [37].

DSA employs two primary pinning strategies to control BCP domain orientation. Chemical pinning utilizes pre-patterned ULs with alternating hydrophobic/hydrophilic (e.g., octadecyltrichlorosilane and polyethylene glycol (PEG) silanes) domains. These patterned surfaces create localized energy wells that thermodynamically stabilize BCP domains at predefined locations, ensuring the precise registration of nanoscale features [38]. Topographic pinning exploits nanoimprinted trenches with depths optimized to ~0.5 times the BCP’s natural periodicity (L_0_), where three-dimensional confinement effects energetically favor specific domain orientations. To further enhance pattern fidelity, silicon dioxide (SiO_2_) trench sidewalls are functionalized with NL brushes, which reduce interfacial energy mismatches and suppress defect formation [24]. These dual strategies of chemical guidance through surface energy modulation and physical confinement through topographic templates synergistically overcome placement accuracy limitations in complex architectures, enabling sub-5 nm lithographic resolutions.

### 3.3. BCP Material

BCPs, particularly di-block copolymers, serve as the foundational material for DSA, an emerging next-generation lithography technique. A di-block copolymer consists of two chemically distinct polymer blocks (e.g., PS-b-PMMA) linked via a covalent bond at their junction. When thermally annealed or exposed to solvent vapor, these polymers undergo microphase separation, driven by the thermodynamic incompatibility between the blocks quantified by χ.

In DSA, BCPs are guided by pre-patterned substrates to self-assemble into well-ordered nanodomains with minimal defects, enabling high-resolution lithographic patterning. Achieving high interfacial energy contrast between the blocks is critical to ensure sharp phase separation and reduced line-edge roughness (LER), which directly impacts pattern fidelity. However, key challenges remain in synthesizing BCPs with low polydispersity indices (PDI < 1.1), high χ values (χ > 0.1), and sufficient etch selectivity, which are critical for pattern transfer to substrates. Recent advances focus on developing high-χ BCPs (e.g., silicon-containing polymers) to achieve sub-10 nm features, although balancing χ with process compatibility (e.g., annealing kinetics, solvent resistance) remains a significant challenge. High-χ materials (χ > 0.2 at 150 °C) like PS-b-PDMS enable sub-10 nm features but require advanced etch selectivity enhancement techniques (Table 1) [39,40]. Emerging material systems, such as poly(ethylene oxide)-block-polybutadiene (PEO-b-PB), have demonstrated potential for achieving sub-5 nm resolutions [25].

As reported by Tokyo Ohka Kogyo (TOK) researchers, a novel series of BCPs has been developed that both achieve sub-10 nm and perpendicular orientations under the same conditions as PS-b-PMMA. The high chi BCP shows the fingerprint and random hole under the same conditions as PS-b-PMMA [35].

## 4. Pattern Transfer Techniques

One of the biggest challenges in the DSA process is pattern transfer fidelity, where the self-assembled BCP pattern is transferred into the underlying substrate layers. Two primary methods for pattern transfer are widely used, plasma-based dry etching and solution-phase wet etching, both of them demonstrating distinct resolution–etch rate tradeoffs.

### 4.1. Dry Etching

Dry etching, particularly reactive ion etching (RIE), is the most common pattern transfer technique in DSA. It involves using plasma to selectively remove one block between the two polymer blocks in a BCP. For example, the PS-b-PMMA BCP film self-assembles on a substrate [41]. The PMMA block is preferentially selectively removed using O_2_ plasma, leaving a PS block. The PS template acts as a hard mask during dry etching. The etch selectivity between PS and the underlying material (e.g., silicon nitride) is critical. High selectivity ensures that the pattern is transferred accurately without significant mask erosion. Key process parameters include plasma composition, ion energy, and chamber pressure. Dry etching offers high resolution, excellent anisotropy, and compatibility with existing semiconductor manufacturing processes. However, achieving high etch selectivity can be difficult, especially for thin BCP films (<20 nm). Additionally, plasma-induced damage to the substrate or underlying layers can occur. 

### 4.2. Wet Etching

**Figure 7:**
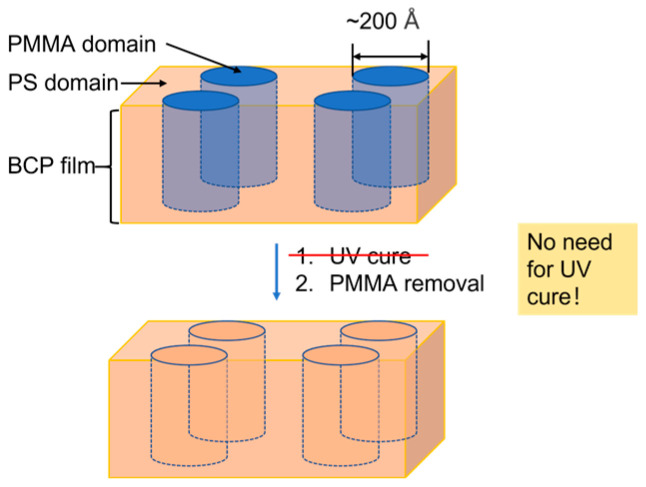
Schematic illustration of UV-free wet etch processing. Adapted from [42].

Wet etching involves using chemical solutions to selectively remove materials, offering advantages such as high etch selectivity and reduced substrate damage. As shown in Figure 7, Wu and Liao demonstrated a wet etch process optimized for DSA patterning in advanced DRAM nodes [42]. They developed a novel process, a one-step facile removal of PMMA by one chemistry, that will certainly eliminate concern with PS surface damage by UV, as well as benefit users with throughput improvement. 

Wet etching offers high selectivity, reducing the risk of mask erosion and substrate damage. It is also compatible with batch processing, improving throughput. However, isotropic etching can limit the resolution and aspect ratio of patterns, making it less suitable for very thin films (<10 nm). Additionally, achieving uniform etching across large areas remains a challenge. As DSA technology continuously develops, wet etching is expected to play an increasingly important role in next-generation nanofabrication.

## 5. DSA Simulation

As previously discussed, DSA has the potential to generate highly ordered nanoscale patterns with sub-10 nm resolutions. DSA simulations are critical to this process, as they help predict the intricate nanoscale pattern formation during DSA. These simulations are key to optimizing the process, ensuring that the desired patterns are achieved with high precision. By utilizing simulations, researchers and engineers can gain a deeper understanding of the underlying physical and chemical mechanisms, which is essential for reducing development time and costs in practical applications.

### 5.1. Core of DSA Simulation Technologies

#### 5.1.1. SCFT: The Precise Predictor of BCP Phase Behavior

Self-consistent field theory (SCFT) is a powerful tool for modeling the phase behavior of BCPs. SCFT treats the BCP system as a collection of interacting polymer chains in a mean-field environment and calculates the self-consistent fields that these chains experience, which ultimately determine the equilibrium morphology of the BCP [43]. SCFT has proven particularly successful in predicting equilibrium morphologies such as lamellae and cylinders. Lamellar structures form when the volume fractions of the two polymer blocks in the BCP are approximately equal, while cylindrical structures arise when one block has a significantly larger volume fraction than the other [44]. The ability of SCFT to accurately predict these morphologies has been demonstrated in numerous studies. For instance, SCFT simulations have been used to study the self-assembly of PS-b-PMMA BCPs, and the predicted lamellar and cylindrical morphologies showed excellent agreement with experimental observations [45]. Furthermore, SCFT’s utility in BCP research has been extended to a more accessible framework for material discovery, making it a widely used approach for investigating a variety of BCP systems [46].

#### 5.1.2. MD: The Dynamic Observer of the Microscopic World

Molecular dynamics (MD) simulation is another important technique for studying BCPs. MD simulations track the time evolution of the positions and velocities of individual atoms or molecules in a system. In the context of BCPs, MD can be used to simulate the intermolecular interactions that drive domain formation and defect dynamics. By applying Newton’s equations of motion to each atom in the system, MD simulation provides a detailed view of the dynamic processes occurring at the nanoscale [47]. For instance, during the formation of BCP domains, MD simulations can show how the polymer chains diffuse and reorganize over time to form the characteristic ordered structures. Additionally, when it comes to defect dynamics, MD can reveal how defects such as grain boundaries and dislocations form, move, and interact with each other. A study in [48] used MD simulations to investigate the self-assembly of BCPs on patterned substrates and observed the formation and evolution of defects during the process, providing valuable insights into improving the quality of DSA-fabricated patterns.

#### 5.1.3. DFT: Quantum Insights for Polymer Design

Density functional theory (DFT) provides atomic-scale insights into block copolymer (BCP) interactions, complementing coarse-grained methods like SCFT and MD. While computationally intensive for large systems, DFT accurately predicts electronic structure, interfacial energetics, and substrate–polymer bonding, which are key factors in directed self-assembly. For example, DFT reveals preferential adsorption of polymer blocks on chemically patterned surfaces, guiding chemoepitaxy optimization. Hybrid DFT/MD approaches now bridge quantum-level precision with mesoscale self-assembly behavior, aiding the design of next-generation BCPs for nanofabrication.

### 5.2. Existing Challenges and Countermeasures

#### 5.2.1. Strategies to Overcome Computational Challenges in DSA Simulations

One of the major challenges in DSA simulations is the high computational cost [49]. Both material simulations, such as SCFT and MD, and lithography simulations often require significant computational resources, due to the large number of atoms or grid points involved in the models and the complex interactions that need to be calculated. The wide variety of materials used in DSA, each with unique physical and chemical properties, adds further complexity. For example, different BCPs have varying chain lengths, compositions, and interaction parameters, while substrates and additives used in DSA processes introduce additional factors that must be accounted for in simulations.

To address these computational challenges, researchers are exploring several strategies. One approach is to develop more efficient algorithms. One effective strategy involves the use of high-performance computing platforms such as supercomputers and graphics processing units (GPUs). GPUs, with their highly parallel architecture, can significantly speed up MD simulations. In this context, the study in [50] proposed a GPU-based open-source SCFT framework that accelerates the simulation of BCP phase behavior, showing a significant improvement in computational speed and efficiency, compared to traditional CPU-based methods.

Machine learning (ML) has also emerged as a promising tool for accelerating block copolymer self-assembly simulations. ML algorithms can rapidly predict the outcomes of complex self-assembly processes by training on large datasets from previous simulations. This reduction in computational time allows for the exploration of a wider design space for process optimization. For example, in the study in [51], researchers applied machine learning methods for template design, effectively controlling the self-assembly of block copolymers to design complex patterns. Additionally, in the study in [52], machine learning models were trained to predict block copolymer self-assembly morphologies, significantly shortening the time required for traditional SCFT simulations, enabling rapid identification of optimized process conditions. This capability was demonstrated in study [53], where the integration of deep neural networks with SCFT simulations was shown to accelerate polymer self-assembly simulations and inverse DSA lithography processes, highlighting the potential of this method in improving the efficiency and accuracy of polymer self-assembly predictions.

#### 5.2.2. Integrated Simulation of Multiple Domains

The integration of material and lithography simulations marks a significant advancement in the design of DSA processes [54,55]. By combining these simulation approaches, researchers can achieve a more comprehensive understanding of the entire DSA process. Material simulations offer insights into the equilibrium nanostructures formed by BCPs, while lithography simulations predict how these nanostructures will be transferred and modified during the lithography steps. This integrated approach enables a more effective optimization of both material properties and lithography process parameters, leading to the production of high-quality patterns with fewer defects.

For instance, Hannon et al. [56] developed an inverse design algorithm that predicts the necessary topographical template to direct the self-assembly of a di-block copolymer to produce a given complex target structure. This approach was optimized by varying the number of topographical posts, post size, and block copolymer volume fraction to yield a template solution that generates the target structure in a reproducible manner. Simulated and experimental results showed very good agreement, demonstrating the effectiveness of integrating material and lithography simulations in DSA process design.

The design and optimization of DSA guiding patterns significantly influence the quality of subsequent DSA patterns. As elucidated in Shim’s study, the guiding pattern formed on the wafer via optical lithography serves as the template for the self-assembly process [57]. The precision of its design, involving aspects such as feature size, pitch, and shape, directly dictates the formation of the final DSA patterns. For instance, a well-optimized feature size can control the initial seed distribution, enabling precise line width control and enhanced integration density in high-density storage applications. However, an inappropriate pitch between guiding patterns may lead to interference during self-assembly, causing pattern misalignment and a decline in chip yield, which is a critical concern in semiconductor manufacturing. Moreover, the book by Shim et al. further emphasizes the importance of a comprehensive approach to guiding pattern design [58]. It highlights that the optimization process should not only focus on geometric parameters but also consider the material properties and process conditions. For example, the shape of the guiding pattern interacts closely with the self-assembly characteristics of the materials used. Regular-shaped guiding patterns tend to produce symmetric DSA patterns, which are suitable for applications demanding high regularity and can reduce stress-induced cracking. In contrast, irregular-shaped patterns offer the potential to create unique topological structures for micro-nano optical devices, yet they require complex algorithms to mitigate distortion risks during self-assembly. Through a combination of inverse DSA and inverse lithography techniques proposed in [57], along with the physical design principles outlined in [58], researchers can iteratively refine the guiding pattern design, thereby enhancing the quality and predictability of DSA patterns in practical applications.

Validating simulation results with experimental data is crucial for ensuring the reliability and accuracy of DSA simulations. However, discrepancies often arise between simulations and experiments due to simplifications in the simulation models, experimental uncertainties, and unforeseen interactions in real-world systems. To address these challenges, researchers are improving simulation models by incorporating more realistic physical and chemical effects. At the same time, advanced experimental techniques are being developed to provide more precise data for validation. For instance, in situ characterization methods, such as scanning probe microscopy (SPM) and synchrotron-based X-ray scattering, offer real-time insights into the nanoscale self-assembly process. The study by Cameron et al. [59] demonstrates how in situ X-ray scattering can be used to track the evolution of nanostructures in BCP thin films, validating SCFT simulations and refining the models to improve their predictive accuracy.

Current simulation technologies, encompassing both material and lithography simulations, have made considerable advancements in enhancing our understanding and prediction of DSA processes. The integration of diverse simulation techniques, coupled with the application of machine learning, has significantly augmented the capabilities of DSA simulations. Looking ahead, one of the primary research challenges will be reducing computational costs without compromising accuracy. Additionally, the development of more general and precise models for a wider range of materials will be essential for further progress. Strengthening the connection between simulations and experimental data through more effective validation methods will also be pivotal for the successful adoption of DSA in industrial applications. With ongoing research and innovation, DSA simulations are poised to play an increasingly critical role in advancing next-generation nanoscale patterning and lithography technologies.

## 6. DSA Industrial Application

DSA is a cutting-edge nanofabrication technique that combines the intrinsic self-assembly properties of BCPs or other molecular systems with external guiding forces to create highly ordered nanostructures. After two decades of development, DSA has made significant advancements in materials and process technologies and has been recognized by IRDS as one of the primary candidate solutions for next-generation lithography. 

Recent advancements highlight DSA’s versatility across silicon (Si)-based and compound semiconductor applications. In CoFeB-based magnetic storage devices, uniform 16 nm critical dimension (CD) arrays with a 27 nm pitch have been achieved, enhancing bit density [60]. For III–V semiconductor nanoelectronics (e.g., InGaAs), hybrid BCP/NIL produced sub-10 nm nanoribbons, enabling multichannel field-effect transistors [61]. For Si-based nanoelectronics, sub-10 nm nanoribbons were fabricated via hybrid BCP/NIL, enabling multichannel field-effect transistors, and similar approaches have been demonstrated for wide-bandgap semiconductors, such as GaN. Sony developed a Si-based back-illuminated CMOS image sensor (BI-CIS), representing the first production-grade report of an image sensor fabricated using the DSA process [62]. Similar potential exists for InP-based photonic devices where DSA enables sub-10 nm photonic crystal patterning. FinFET fabrication studies on silicon-on-insulator (SOI) substrates reveal comparable electrical performances between DSA and self-aligned quadruple patterning (SAQP) at a 27 nm pitch, with DSA reducing mask counts and lithography steps [63,64]. Cost analyses demonstrate a 17% cost reduction in Si-based front-end-of-line (FEOL) processes through batch-processed DSA, driven by reduced thermal annealing times [65]. Currently, research institutions, such as IBM, IMEC, Intel, TEL, and CEA-Leti, have established 300 mm wafer DSA pilot lines, publishing a series of critical research achievements in process development, integration, defect control, and device applications, aiming to achieve industrial implementation in production environments. 

### 6.1. Pattern Rectification (Roughness Improvement)

As reported by Intel researchers, DSA can be applied for EUV line/space resist rectification; DSA fundamentally improves systematic and random variabilities. The LER/LWR of the DSA line/space pattern with a 24 nm pitch is improved by DSA rectification to 1.70/1.40 from 1.88/2.71, respectively [66]. The roughness of line and space patterns was improved by exploring BCP thickness, enhanced UL, adjusting BCP L_0_, and etch selectivity. 

The work reported by EMD concentrated on improving the LER and LWR using high-chi BCPs. Substantial LWR (0.892 nm, ~300%) and LER (1.065 nm, ~175%) improvements were found for PME-3443a (HChi-3a) BCP, compared to EUV (LER/LWR: 1.884 nm/2.71 nm) [67].

### 6.2. Pitch Multiplication for Hexagonal Hole

**Figure 8:**
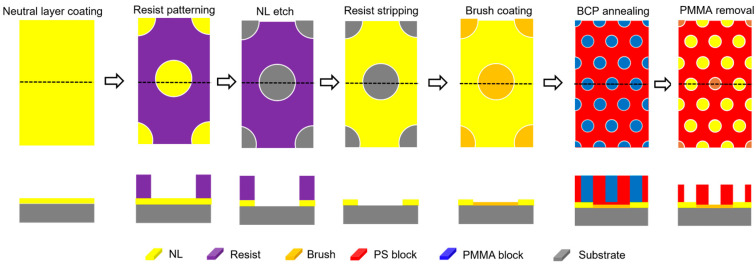
SEM pictures of the hexagonal pattern at each step. Adapted from [69].

DSA technology has emerged as the focus of hole patterning applications, drawing considerable attention from the semiconductor industry. As part of the ongoing push to commercialize DSA technology, TEL partnered to integrate DSA techniques into hole patterning processes. In TEL’s report, 40 nm, 35 nm, and 30 nm pitch hexagonal hole patterns were formed by chemical-epitaxy DSA flow with nine multiplications [68]. It was confirmed that phase separation is possible up to 30 nm in both systems with a multiplication number of nine, which has high stability. Moreover, LCDU and placement error have been improved. As shown in Figure 8, the formation of a DSA hexagonal hole with a relatively long pitch of 50 nm was confirmed in 2025 [69]. The template pattern pitch was 150 nm, and the pitch shrank to 50 nm after the DSA process. The LCDU (3σ) and placement error (3σ) were 1.7 nm and 3.2 nm, respectively. Utilizing chemical epitaxy processes to create high-density hole patterns presents an effective strategy for lowering rising lithography costs.

### 6.3. LCDU and PPE Improvement

Dong-Ook Kim’s study developed a novel high-χ block copolymer (HChi BCP) to optimize the DSA rectification process for EUV-defined contact hole (C/H) patterns [70]. Compared to conventional PS-b-PMMA, the HChi BCP demonstrated significantly improved local CDU from 1.71 nm to 1.41 nm and reduced pattern placement error (PPE) from 3.13 nm to 2.18 nm, compared to PS-b-PMMA baselines.

**Figure 9:**
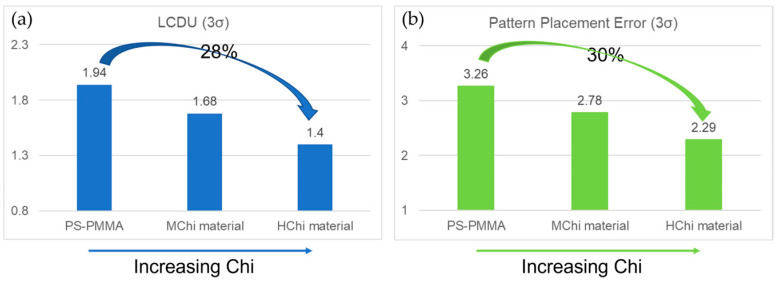
Effects of different BCP adjustments on (**a**) LCDU and (**b**) pattern placement error values. Data from [71].

Research by Xianfeng Gao developed a series of BCP materials with different chi values and modifications [71]. The results demonstrate that first-generation PS-b-PMMA BCPs exhibit constrained performance at larger pitch (L_0_ ~ 50nm) sizes due to their slower system kinetics. Moderate chi (MChi)-modified BCPs improve the kinetics of large Mn PS-b-PMMA materials. Meanwhile, additives in MChi BCPs can help mitigate the defects of DSA. Compared with PS-b-PMMA, the LCDU and PPE of (HChi) materials were reduced by 28% and 30% (Figure 9). High-chi (HChi) materials demonstrated improved roughness and uniformity. High-chi (HChi) materials hold great promise for small-pitch applications in the manufacturing of future DRAM devices.

## 7. DSA Challenges

Despite its potential as a next-generation lithography alternative, DSA faces significant technical and industrial obstacles that must be resolved to enable widespread application.

### 7.1. BCP Material Complexity

One primary challenge is the material complexity in BCP engineering. DSA relies on precisely engineered BCPs to achieve nanoscale self-assembly, requiring meticulous control over polymer composition, molecular weight, and interfacial interactions. Minor deviations in material properties, such as polydispersity or contamination, can lead to defects like dislocation, bridging, or incomplete phase separation, directly impacting pattern fidelity. Additionally, synthesizing BCPs with tailored properties for sub-20 nm node applications remains costly and time-consuming, hindering its large-scale application.

### 7.2. DSA Defect Formation and Control

**Figure 10:**
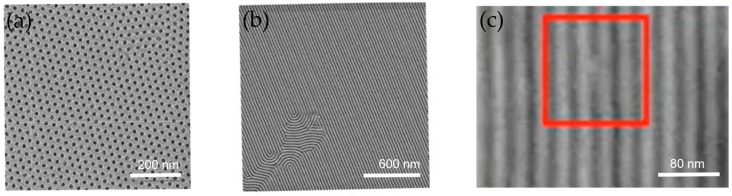
Typical defect types in DSA: (**a**, **b**) dislocations and (**c**) bridging in the red square.

Defect density remains the primary barrier to DSA application, with current benchmarks (~10/cm^2^) far exceeding industry standards (<1/cm^2^). Figure 10 classifies critical DSA defects and the key defect types. (1) Dislocations are unique to DSA, arising from BCP phase misalignment and are irreparable during etching. (2) Bridge defects are shared with conventional lithography but are persistent due to material and process mismatches.

The key factors affecting the occurrence of defects include material and process aspects. Material aspects, including BCP purity, molecular weight homogeneity, and interfacial energy matching between neutral and guiding layers, significantly influence defect rates, where hydrophobicity mismatches in guiding layers directly exacerbate dislocation density formation [72,73].

Thermal annealing parameters (time/temperature) critically govern defect annihilation kinetics. While extended annealing time reduces dislocation densities, practical time limits their efficacy [8]. Additionally, film thickness exhibits a dual role, with thinner BCP layers (<35 nm) suppressing bridging defects but enhancing dislocation risks [74].

Recent advancements reduce defects on the DSA process through two primary strategies: (1) surface modification of guiding layers via plasma treatment or brush coatings enhances interfacial energy matching [72], and (2) optimized guiding layer topography minimizes height disparities, thereby reducing bridge formation.

### 7.3. DSA Metrology Challenges and Solutions

DSA metrology faces dual challenges of insufficient chemical contrast during pre-etch evaluation and throughput constraints in defect inspection [8]. To resolve these limitations, three primary strategies have been adopted: (1) post-etch optical scatterometry, which boosts dislocation detection sensitivity tenfold through phase-sensitive analysis [75]; (2) dark-field microscopy for the precise identification of bridging defects in pattern-transferred substrates; and (3) optimized etch depth schemes that magnify defect signatures via controlled topography modulation. Although scanning electron microscopy (SEM) remains the industry-standard metrology for nanoscale resolution, its restricted imaging area (~100 μm^2^) needs integration with optical techniques for comprehensive wafer-scale defect mapping. Recent advancements in hyperspectral scatterometry systems now enable rapid, non-destructive quantification of dislocation densities across 300 mm wafers, achieving <0.1% false detection rates through machine learning-enhanced signal processing.

Despite these challenges, DSA remains recognized as a promising hole patterning technology, demonstrating potential to enhance pattern uniformity and to solve patterning challenges at sub-30 nm and even sub-20 nm scales.

## 8. Conclusions and Outlook

DSA lithography is an advanced patterning technology that combines "bottom-up" self-assembly of BCP thin films with “top-down” lithographic techniques, such as optical or EBL, to fabricate high-resolution nanostructures. This review provides a comprehensive overview of DSA’s principles, materials, processes, applications, and challenges in industrialization. DSA offers advantages such as reduced manufacturing costs, CD shrinkage, pattern density multiplication (e.g., hole and line patterns), and improved roughness of lines and contact holes, thereby overcoming the limitations of conventional lithography. Furthermore, when combined with common lithography techniques, including EUV, DUV, EBL and NIL, DSA enhances pattern resolution, repairs defects, and improves CDU. These capabilities have enabled its widespread application in silicon logic devices, GaN-based power memory, Si-based CMOS image sensors, and InP-based photonic devices. However, achieving precise control of BCP composition and reducing defect density remain arduous challenges. Additionally, there is an urgent need to establish an integrated DSA-based patterning ecosystem including materials, equipment, processes, computational lithography, simulation tools, and electronic design automation (EDA) to promote DSA into full-scale industrial production.

Looking ahead, the future of DSA lies in the development of advanced BCP materials with tailored properties, DSA-specific simulation software (e.g., Synopsys S-Litho™, version V-2024.06; siSCFT from Shanghai Institute of Optics and Fine Mechanics, version 1.0), novel self-assembly methods, and hybrid strategies combining DSA with next-generation lithography. Future DSA advancements must prioritize scale-dependent material–process co-optimization, where sub-10 nm patterning demands high-χ BCPs and defect-tolerant processes, while scaling beyond 50 nm may require hybrid DSA approaches integrating colloidal self-assembly. These innovations will enable precise control over DSA to achieve higher-resolution patterns. With the development of the BCP film and DSA, it is poised to become one of the most promising advanced lithography techniques, narrowing the gap between nanoscale fabrication demands and the limitations of current technologies. By facilitating interdisciplinary collaboration across materials science, process engineering, and computational modeling, DSA is expected to drive breakthroughs in semiconductor manufacturing and beyond, finally reshaping the situation of nanotechnology.

## Figures and Tables

**Table 1:** Key BCP families and their properties. Data from [39,40].

Polymer System	χ Parameter	Feature Size (L_0_)	Applications
PS-b-PMMA	0.03–0.05	15–30 nm	Lamellae for line/space
Polystyrene-b-Poly(2-vinylpyridine) (PS-b-P2VP)	0.10–0.15	8–15 nm	Metal nanowire templates
Polystyrene-b-Polydimethylsioxane (PS-b-PDMS)	0.25–0.35	5–10 nm	High-χ sub-10 nm patterns
Poly(t-butyl acrylate)-block-poly(methyl methacrylate) (PtBA-b-PMMA)	0.08–0.12	10–20 nm	Solvent-annealed vertical pores

## Data Availability

No new data were created in this study.
